# The Serotype Distribution among Healthy Carriers before Vaccination Is Essential for Predicting the Impact of Pneumococcal Conjugate Vaccine on Invasive Disease

**DOI:** 10.1371/journal.pcbi.1004173

**Published:** 2015-04-16

**Authors:** Stefan Flasche, Olivier Le Polain de Waroux, Katherine L. O’Brien, W. John Edmunds

**Affiliations:** 1 London School of Hygiene and Tropical Medicine, London, United Kingdom; 2 International Vaccine Access Center, Johns Hopkins Bloomberg School of Public Health, Baltimore, Maryland, United States of America; Yale School of Public Health, UNITED STATES

## Abstract

Pneumococcal conjugate vaccines (PCVs) have substantially reduced morbidity and mortality of pneumococcal disease. The impact of the 7-valent PCV on all-serotype invasive pneumococcal disease (IPD) among children was reported to vary between high-income countries. We investigate the ability to predict this heterogeneity from pre-vaccination data. We propose a parsimonious model that predicts the impact of PCVs from the odds of vaccine serotype (VT) among carriers and IPD cases in the pre-PCV period, assuming that VT are eliminated in a mature PCV programme, that full serotype replacement occurs in carriage and that invasiveness of the NVT group is unchanged. We test model performance against the reported impact of PCV7 on childhood IPD in high-income countries from a recent meta-analysis. The odds of pre-PCV7 VT IPD, PCV schedule, PCV coverage and whether a catch up campaign was used for introduction was gathered from the same analysis. We conducted a literature review and meta-analysis to obtain the odds of pre-PCV7 VT carriage in the respective settings. The model predicted the reported impact on childhood IPD of mature PCV programmes; the ratio of predicted and observed incidence risk ratios was close to 1 in all settings. In the high income settings studied differences in schedule, coverage, and catch up campaigns were not associated with the observed heterogeneity in impact of PCV7 on childhood all-serotype IPD. The pre-PCV7 proportion of VT IPD alone also had limited predictive value. The pre-PCV7 proportion of VT carriage and IPD are the main determinants for the impact of PCV7 on childhood IPD and can be combined in a simple model to provide predictions of the vaccine preventable burden of IPD.

## Author Summary

Pneumococcal vaccines (PCVs) that protect children against 7, 10 and 13 of the most pathogenic pneumococcal serotypes have substantially reduced childhood morbidity and mortality. A recent analysis that evaluated the impact of the 7 valent PCV in multiple high income settings in North America, Europe and Oceania found that the magnitude of all-serotype invasive pneumococcal disease reduction varied greatly between settings (24%-83%). We explored potential sources for that variation, including differences in disease epidemiology before vaccination, vaccine coverage, vaccine schedules and the use of catch-up campaigns for introduction. We find that differences in reported disease impact among mature PCV programmes are likely to be unrelated to the differences in the vaccine programme but can be predicted from a simple model based on pre-vaccination epidemiology, in particular the proportion of vaccine serotypes detected among patients with invasive pneumococcal disease and the proportion of vaccine serotypes that are found in the nasopharynx of healthy individuals. This model presents a useful tool to estimate the potential impact of PCVs (as a relative rate reduction), highlights the essential role of pre-vaccination carriage in healthy individuals for disease impact of PCVs and can estimate the prevented burden of disease where disease surveillance is unavailable.

## Introduction

The Word Health Organisation estimates that *Streptococcus pneumoniae* is associated with about 5% of all-cause child mortality globally; over 90% of these pneumococcal deaths occur in low income countries [1]. Pneumococcal conjugate vaccines (PCVs) are part of the routine infant immunization schedule in most high income countries, resulting in a substantially reduced burden of serious pneumococcal disease [2–4]. PCVs are also being introduced into the routine vaccination programmes of low and middle income countries, partly with the financial support of Gavi, the Vaccine Alliance [5–7]. PCVs provide protection against nasopharyngeal carriage and disease for serotypes included in the vaccine (VT); these serotypes have been associated with the majority of invasive pneumococcal disease (IPD) globally [8]. Protection against VT nasopharyngeal carriage opens an ecological niche which is filled by the non-vaccine pneumococcal serotypes (NVT); a process termed serotype replacement [9–11]. This increase in NVT colonization prevalence results in an increased rate of NVT disease; however, because these serotypes are inherently less likely to cause disease among young children than VT strains, there is a substantial net benefit [12]. Understanding the interplay between VT protection and NVT replacement is essential for the assessment of the total impact of PCVs [13,14].

Despite being consistently beneficial, substantial heterogeneity in the relative impact of PCV7 on all-serotype IPD rates has been observed across settings, with impact estimates in children younger than 5 years ranging from 24% to 83% in mature programmes [12]. This heterogeneity is thought to result from interactions of vaccine coverage, vaccination schedule, serotype distribution, demographic structure and social mixing patterns, catch up campaigns at introduction, time since PCV introduction, and disease surveillance sensitivity. The contribution of each of those factors to the observed heterogeneity in PCV impact on all-serotype IPD is unclear. PCVs are amongst the most expensive vaccines that are routinely used for infant vaccination. Although the Advance Market Commitment (AMC) and the support of Gavi, the Vaccine Alliance substantially reduced the PCV price for low income countries [15] and pooled procurement might help reducing the costs for middle income countries [16] PCVs pose a considerable investment that requires robust evidence about its likely impact. A better understanding of the main factors that determine the impact of PCVs is essential to reduce the uncertainty around the impact and cost-effectiveness estimates of PCVs in PCV-naive countries, as well as for the assessment of the likely impact of future PCV compositions and to inform programme maintenance justifications. While better impact predictions may help with a faster introduction of PCVs globally, the justification of existing pneumococcal immunisation programmes will become particularly important for countries that have introduced PCVs with financial support from Gavi, the Vaccine Alliance and will graduate from that support. These countries have to evaluate the merits of vaccination at or below the agreed tail price of PCV under the AMC agreements, however, measuring disease impact is only possible in a limited number of countries. Disease impact models are therefore important for many countries.

Available methods to predict the likely impact of PCV on disease include models accounting for carriage and disease serotype distribution and replacement as the main drivers for PCV impact [14,17,18] and more complex transmission models [19–21]. To date little validation of the models capability to accurately predict post vaccination changes in pneumococcal disease is available. We evaluate the ability of the pre-vaccination pneumococcal serotype distribution in both nasopharyngeal carriage and disease, vaccine coverage, schedule and catch up campaigns to predict the impact of PCV7 on invasive pneumococcal disease in children less than five years old and the importance of each of those factors for the accuracy of the prediction.

## Materials and Methods

### A predictor of PCV impact on pneumococcal disease

To predict the impact of PCV on pneumococcal disease we employ a model that is similar to previous approaches and uses changes in pneumococcal carriage to predict the impact of PCV on IPD [14,17,18,22].

For simplicity we assume a perfectly monitored homogenous population. Note that the methods can be derived similarly if imperfect sensitivity of carriage and/or disease surveillance and a heterogeneous population is assumed, as long as surveillance sensitivity and population heterogeneity does not change after vaccination. In this population and in the absence of PCV vaccination the rate of pneumococcal disease (*D*) per person-time can be expressed as a function of the rate of carriers (*C*) per person-time and the average ratio at which a carriage episode results in disease (Φ = D/C), stratified by vaccine and non-vaccine serotypes respectively:

$$D\;=\;C_{vt}\Phi_{vt}+\;C_{nvt}\Phi_{nvt}.\;$$

The parameter Φ is also called the case to carrier or invasiveness ratio for VT or NVT, which corresponds to the mean of the serotype specific invasiveness ratios weighted by serotype-specific carriage prevalence. We further assume that (i) vaccine serotype carriage will eventually be eliminated through routine use of PCV ($C_{vt}^{*}\;=\;0$), the superscript star indicating post vaccination, (ii) that a proportion, λ∈[0,1] of pre-PCV VT carriage is replaced by NVT carriage ($C_{nvt}^{*}\;=\;{\lambda C}_{vt}+C_{nvt}$), and (iii) that the invasiveness ratio of the non-vaccine serotypes group remains unchanged after vaccination ($\Phi_{nvt}^{*}\;=\;\Phi_{nvt}$). Then the rate of pneumococcal disease in a mature PCV-vaccination programme (D*), when the programme has been in place long enough for direct and indirect effects to become fully established, can be expressed as a function of pre-vaccination pneumococcal carriage rates and the invasiveness of the NVT group of serotypes:

$$D^{*}\;=\;\left({\lambda C}_{vt}+C_{nvt}\right)\Phi_{nvt}.\;$$

Then the expected Incidence Rate Ratio (IRR), simplifies to:

$$IRR\;=\;\frac{D^{*}}{D}\;=\;\frac{\lambda c+1}{d+1},\;$$

where c and d are the odds of VT carriage and disease, respectively prior to vaccination. That is *c* = *C*_*vt*_ / *C*_*nvt*_ and d = *D*_*vt*_ / *D*_*nvt*_. Note that, although technically *c* and *d* are the odds based on disease and carriage rates, it is equivalent to calculate the odds based on counts even if carriage and disease data arise from samples of different sizes or proportions of VT and NVT among pneumococcal carriage and disease rates. For convenience we will mainly refer to proportions hereafter.

Therefore, if *c* and *d* can be obtained from representative samples of the population, the expected percentage change in IPD after vaccination, one of the key measures of vaccine impact, can be estimated from pre-vaccination data alone (given an informed assumption on the level of replacement (*λ*) is available).

### Data for the validation of the prediction model

To test the performance of our predictor and the potential importance of other factors in accounting for the observed heterogeneity of PCV impact we compared the change in IPD rate after routine use of PCV7 with model predictions that use pre-vaccination data on the proportion of VT in IPD and pneumococcal carriage in a sample of the population from the same study site or country. We assumed that carriage and disease in these sub-population were representative of that in the respective study site or country. We further assumed that the mean duration of carriage for VT and NVT is similar which allowed the use of carriage prevalence for the calculation of the odds of VT carriage. We studied two predictions: (i) our main prediction that assumes complete serotype replacement in nasopharyngeal carriage (*λ* = 1) as is observed in most settings where PCV7 was introduced for routine vaccination and (ii) an alternative prediction that assumes no serotype replacement in carriage (*λ = 0*). This prediction requires no carriage data because it reduces to $IRR\;=\;\frac{D_{nvt}}{D}$. This prediction illustrates the impact of assuming that all vaccine preventable IPD is eliminated and does not take into account serotype replacement

#### Observed IRR

The impact of 7-valent pneumococcal conjugate vaccination on invasive pneumococcal disease, as measured by incidence rate ratios (IRR), has recently been summarized from country specific IPD disease rate surveillance data pre- and post- PCV routine use [12]. We used the IRR estimates on all serotype IPD in children under 5 years of age three years after the start of routine vaccination as the observed impact against which we assessed the predicted impact and the role of potential modifying factors on those estimates (see Table 1). Three years has been deemed sufficient time to allow for almost complete elimination of VT disease and completion of serotype replacement in carriage and disease in under 5 year old children in the UK and US [4,23]. This may vary in other countries, depending on factors including vaccine coverage and the use of catch up campaigns. However, Feikin and colleagues [12] reported a reduction of VT IPD in under 5 year old children that was significantly lower than 90% in the 3 years after introduction of vaccination in only 1 out of 14 settings. The mean reduction after 3 years was 91%. Sensitivity analyses which compare model predictions against observed impact after 4, 5 and 6 years are reported in the appendix. Only data in under 5 year old children were considered because carriage information in older age groups is sparse and indirect effects, both VT elimination and serotype replacement, in those age groups, take longer to reach maximum impact. To limit the potential bias of surveillance artefacts we excluded all settings that reported a decrease of NVT IPD incidence after the introduction of PCV. Hence Norway was excluded from the main analysis but was included in our assessment of model sensitivity and reported in the appendix.

**Table 1:** Description of the data.

	Vaccine schedule	Catch up	PCV7 coverage*	Sites	VT%	N IPD	Sites	VT%	N PNC	Source	Observed	Predicted (*λ = 1*)	Predicted / Observed
				Invasive Pneumococcal Disease			Pneumococcal Carriage				IRR		
ABCs	3+1	Y	39%	Active Bacterial Core surveillance USA	88%	715	Atlanta	61.5%	91	[45]	0.33 (0.27 to 0.41)	0.31 (0.23 to 0.44)	0.95 (0.64 to 1.41)
AIP	3+1	Y	46%	Alaska	81%	95	8 rural villages	55.4%	377	[46]			
							Anchorage	53.8%	171	[47]			
							Pooled estimate	54% (25% to 77%)			0.40 (0.22 to 0.73)	0.41 (0.21 to 0.87)	1.01 (0.42 to 2.68)
AUSI	3+PPV	Y	77%	Australia Indigenous	66%	100	Remote community in northern Australia	30.0%	80	[48]	0.60 (0.29 to 1.22)	0.49 (0.35 to 0.65)	0.80 (0.37 to 1.72)
AUSN	3+0	Y	90%	Australia non- Indigenous	89%	1245	Darwin	80.8%	125	[48]	0.46 (38 to 0.57)	0.57 (0.40 to 0.90)	1.25 (0.81 to 2.05)
DEN	2+1	Y	89%	Denmark	70%	455	Roskilde	33.2%	247	[49]	0.39 (0.27 to 0.57)	0.45 (0.38 to 0.53)	1.15 (0.77 to 1.73)
E&W	2+1	Y	89%	England & Wales	78%	3450	Hertfordshire	67.1%	85	[9]			
							Oxford	56.4%	349	[50]			
							London	57.1%	119	[51]			
							Sheffield	68.5%	111	[52]			
							Oxfordshire	56.7%	60	[53]			
							Oxfordshire	64.2%	95	[54]			
							Pooled estimate	61% (54% to 68%			0.55 (0.50 to 0.61)	0.57 (0.48 to 0.69)	1.03 (0.85 to 1.30)
NAV	3+1	Y	68%	Navajo and White Mountain Apaches	58%	100	Navajo and White Mountain Apaches	38.0%	258	[55]	0.76 (0.38 to 1.28)	0.68 (0.52 to 0.85)	0.89 (0.42 to 1.86)
NCK	3+1	Y	47%	Northern California Kaiser Permanente	81%	88	California	60.0%	5	[56]	0.70 (0.38 to 1.53)	0.48 (0.19 to 204.55)	0.71 (0.22 to 2.45)
NLD	3+1	N	94%	Netherlands	74%	245	Western Netherlands	55.4%	213	[57]			
							Rotterdam	54.5%	220	[58]			
							Pooled estimate	52% (21% to 79%)			0.53 (0.33 to 0.86)	0.56 (0.33 to 1.17)	1.06 (0.53 to 2.45)

Description of study sites contributing information about pre-PCV IPD cases [12] and NP colonization characteristics, in children less than 5 years of age. Where multiple studies on nasopharyngeal carriage per IPD setting were included also a pooled estimate (S1 Fig) is presented.

*average vaccine coverage in the first 3 years after introduction of PCV7

#### Pre-vaccination IPD serotype distribution data

In addition to the observed impact of PCV7 on IPD, Feikin et al. [12] also reported the pre vaccination proportion of VT IPD for each surveillance setting (see Table 1). The proportion of NVT IPD was calculated as 1-proportion of VT IPD.

#### Pre-vaccination carriage serotype distribution data

Studies with carriage prevalence estimates for those settings where the impact of PCV7 on IPD was reported, or any subset of that population, were identified through a systematic review, based on the citations retrieved in a recent systematic review of nasopharyngeal carriage in adults and children [24] (Table 1 and S1 Fig). In brief: MEDLINE and Embase electronic databases were used to retrieve articles up to 23rd August 2013 (i.e. week 35) employing the following combination of search terms: ‘(pneumonia OR pneumoniae OR pneumococcal OR pneumococcus) AND (carriage OR colonization OR colonisation)’ in the title or the keywords or the abstract. Articles which fulfilled the eligibility criteria by providing (i) pneumococcal nasopharyngeal carriage prevalence estimates (ii) in a population not previously exposed to PCV, with (iii) nasopharyngeal sampling and transport procedures as well as *S*. *pneumoniae* culture based on WHO guidelines and (iv) where the study was not restricted to specific serotypes or to *S*.*pneumoniae* with specific patterns of antibiotic sensitivity were retrieved.

Of the 376 studies that provided pre-PCV NP carriage estimates, 360 were excluded because they were not in populations where the disease impact was monitored, or because they did not report the proportion of under 5 year old children with nasopharyngeal carriage of any of the serotypes included in PCV7. 16 studies provided data on carriage serotype distribution before introduction of PCVs (Table 1). The carriage data were extracted by two reviewers independently and discrepancies were resolved by consensus.

We abstracted information on the number of carriers of encapsulated pneumococci (assuming that vaccination has no impact on non-encapsulated pneumococci and that those do not substantially contribute to serotype replacement) and the proportion carrying serotypes included in PCV7. In longitudinal studies with repeated sampling of the same individuals, the average VT and NVT carriage prevalence was calculated and the study size was assumed to be the number of unique study participants. We did not account for differences in age distribution due to non-random sampling from the under 5 year old population. Each of the included carriage studies was conducted only on a small subset of the respective populations monitored for IPD. We assumed that the carriage serotype distributions in those studies are representative of the carriage serotype distributions found in the populations that were monitored for IPD.

Where multiple studies on nasopharyngeal carriage were conducted within different subsets of the same population that were monitored for IPD, the results from those studies were combined via a Bayesian random effects meta-analysis.

#### Data on other potential sources of heterogeneity in PCV impact

Feikin et al. [12] reported information on potential sources of heterogeneity for the impact of vaccination including the average vaccine coverage in the first 3 years after introduction of PCV7, the routine vaccination schedule and whether PCV was introduced with a catch up campaign among older children (Table 1).

### Statistical analysis

To calculate the predicted IRRs and its corresponding distributions we assumed that both the proportion of VT among carriers and among IPD were samples from binomial distributions and drew 10,000 bootstrap samples. Where the proportion of VT carriers was derived through the Bayesian meta-analysis we drew the bootstrap samples from the respective posterior distribution instead (S2 Fig). Similarly, we assumed that the observed IRR were samples from log-normal distributions with confidence bounds matching those reported by Feikin et al [12].

We calculated the marginal distribution of observed IRRs for a specific schedule, coverage range or the use of a catch-up campaign upon implementation by bootstrap sampling from the respective observed IRR distributions (S3 and S4 Figs). The different impact of, e.g. a 3+1 schedule versus a 2+1 schedule, on the observed IRR was estimated through the ratio of the marginal distribution of IRRs of the 3+1 schedule settings and the marginal distribution of IRRs of the 2+1 schedule settings. A ratio centered around 1 indicates that the average impact of PCV7 in settings with either a 2+1 or a 3+1 schedule was similar.

We estimated the setting specific performance of our predicted IRRs by calculating the ratio of the predicted IRR to the observed IRR. A ratio of 1 is indicative of a perfect prediction. Non-parametric bootstrapping methods were used to infer confidence intervals.

All analysis was performed in R version 3.1 [25].

## Results

The summary analysis of PCV impact on IPD three years after introduction of PCV7 [12] included data from 13 sites that met the inclusion criteria: indigenous and non-indigenous Australia, Calgary, Denmark, England and Wales, Crete, the Netherlands, Scotland, Switzerland, the US general population (Active Bacterial Core Surveillance), Alaska, Navajo Nation and Northern California (Kaiser Permanente). We identified childhood NP carriage information stratified by VT and NVT from the pre-PCV era from 9 of the 13 sites (Table 1). No carriage information was available in any healthy subpopulation of Calgary, Crete, Scotland and Switzerland. In England and Wales, Netherlands and Alaska more than one carriage study was identified. There was little or moderate heterogeneity between the study estimates of VT carriage proportion within the different sub populations of the same setting. Respective studies were pooled through Bayesian random effects meta-analysis to provide a single estimate of the proportion of VT among carriers for each setting (Table 1 and S2 Fig).

**Fig 1:**
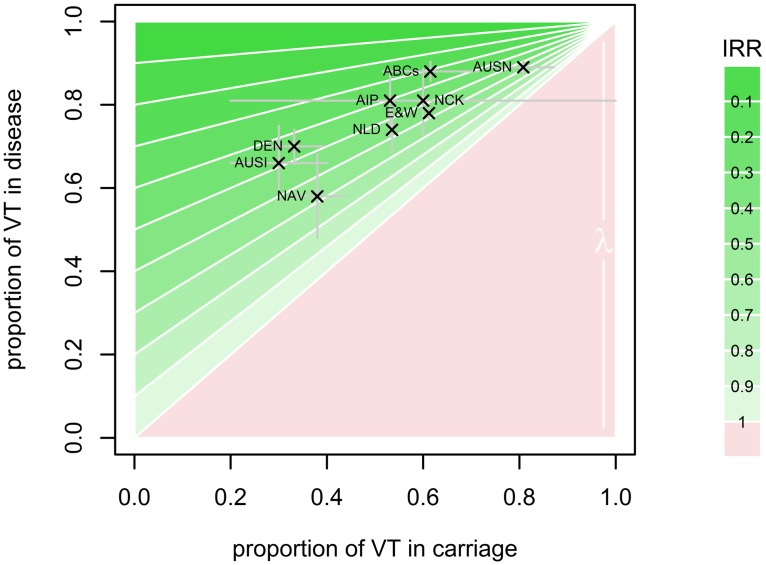
Overview of the impact of model parameterisation on the predictions. The predicted IRRs in pneumococcal disease are shown in dependence of the proportions of VTs in carriage and IPD before vaccination. Shades of green represent predicted IRRs corresponding to a predicted reduction in disease. The height of the red triangle is determined by the extent of serotype replacement (shown for *λ =* 1). The data on pre-vaccination PCV7 VT proportions in carriage and disease from the setting listed in Table 1 and their respective binomial confidence bounds are superimposed to illustrate the effect of differences in serotype distribution in both carriage and disease on the predicted impact of vaccination on pneumococcal disease.

Fig. 1 illustrates how the serotype distributions in pneumococcal carriage and disease shape the predicted incidence risk ratios in the prediction model: the higher the proportion of VT in disease, the higher the predicted impact (lower IRR) and the lower the proportion of VT in carriage the higher the predicted impact. In particular this shows how serotype replacement in nasopharyngeal carriage and differences in serotype distribution in carriage prior to vaccine introduction can lead to vastly different vaccine impact predictions in two settings with similar contribution of VT to the pneumococcal disease burden.

There was little difference between the average observed impact of PCV7 between settings with either different schedules or different coverage levels (S3 and S4 Figs). The impact in settings using a 2+1 schedule was slightly higher than in 3+1 schedule settings and the impact in 3+0 and 3+1 schedule settings was similar; ratio of IRRs 0.87 (0.20 to 2.91) and 1.00 (0.24 to 3.61). In settings with an average vaccine coverage of under 70% and those with a coverage between 70% to 90% the impact of PCV7 was similar to the impact in settings where vaccine coverage had been over 90%; ratio of IRRs 1.00 (0.39 to 2.42) and 0.95 (0.26 to 4.18). Settings which used a catch-up campaign for introduction of PCV7 reported on average a 25% (-65% to 325%) higher impact than those which had not.

**Fig 2:**
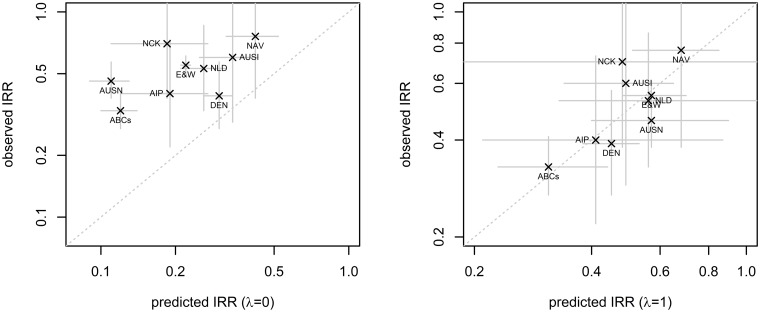
Model predictions in comparison to observed IRR. Comparison of predicted and observed impact of PCV7 on all-serotype IPD in children younger than 5 years, including confidence intervals, assuming no serotype replacement (left panel) or full serotype replacement (right panel) of VT carriage with NVT carriage. ABCs, AIP, AUSI, AUSN, DEN, E&W, NAV, NCK and NLD represent the Active Bacterial Core surveillance USA, Alaska (Calgary), Australia Indigenous, Australia non- Indigenous, Denmark, England & Wales, Navajo and White Mountain Apaches, Northern California Kaiser Permanente and Netherlands respectively.

**Fig 3:**
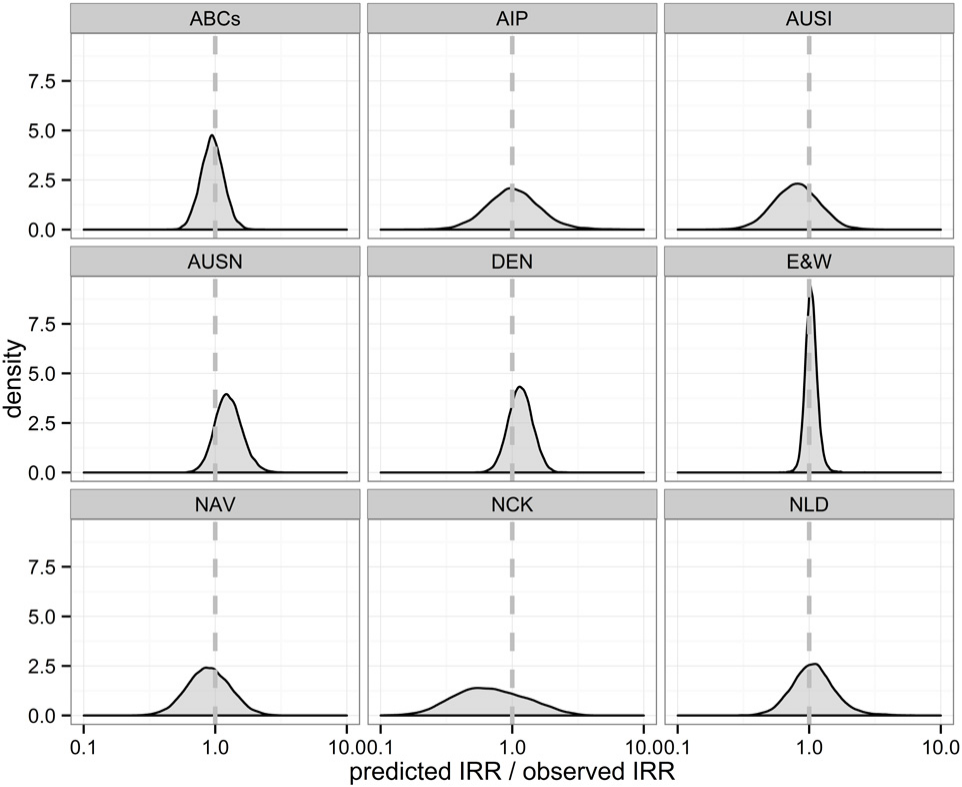
Model validation. Distributions of the ratio of predicted and observed IRR for each setting assuming full serotype replacement. A value of 1 represents perfect agreement between prediction and observation.

Assuming no serotype replacement in carriage (*λ = 0*) for the prediction led to consistent overestimation of vaccine impact (Fig. 2). With the assumption of complete serotype replacement in carriage (*λ =* 1), however, we were able to closely predict the impact of routine PCV7 use on paediatric IPD 3 years after introduction (Fig. 2 and Fig. 3). The corresponding ratios of predicted and observed IRRs are provided in Table 1.

## Discussion

Routine pneumococcal conjugate vaccination has led to sustained reductions of all-serotype invasive pneumococcal disease in children, albeit of varying absolute and relative magnitudes across various countries and surveillance sites. Here we aimed to understand the factors related to the observed heterogeneity of PCV7 impact on IPD in children under five years of age 3 years after the start of routine vaccination so that estimates of that impact could reliably be produced for countries without disease impact data. Our analysis shows that the proportion of VT-IPD in the pre-PCV period, which is sometimes used as a measure of the potential vaccine preventable burden of *S*. *pneumoniae*, is consistently overestimating the observed impact of PCVs on overall IPD because it ignores the effect of serotype replacement. However, when supplemented by the odds of VT carriage pre-PCV in the same population our proposed model, assuming full serotype replacement in carriage, is highly predictive for the observed relative impact (IRR) of PCVs on overall IPD.

We find that in the studied sites neither differences in schedule nor vaccine coverage substantially contributed to the heterogeneity of IPD impact observed from surveillance three years after the introduction of PCV7. While there is little doubt that sufficient vaccine coverage is essential for the success of a PCV programme we find that even in those settings where the average vaccine coverage during the first 3 years after implementation was below 70% the observed impact of vaccination was similar to settings with higher coverage. This may be due to the strong herd protection induced by PCV7 in high income countries, even at low coverage levels, which has helped to control VT transmission and as a consequence VT IPD. We find some evidence of a higher impact of vaccination in settings that introduced PCV with a catch-up campaign, albeit with substantial heterogeneity of PCV impact between those settings.

The method for prediction of the impact of PCV7 builds on the idea that, because of serotype replacement, the case to carrier ratio of serotypes or serotype groups is an important determinant for the success of pneumococcal conjugate vaccines, as has been proposed earlier in similar model approaches [9,14,17,18,22,26]. A model using the inherent link of carriage and IPD has been proposed for monitoring IPD through post PCV nasopharyngeal carriage in the absence of IPD surveillance and has been shown to provide valid predictions for most of the 5 studied sites [18]. Similar models have used pre-vaccination carriage prevalence and IPD incidence to predict the impact of pneumococcal vaccines on IPD. One was validated against a single estimate of IPD impact from US ABC data [22]. We present here a minimalistic tool that utilises information on the serotype distribution of carriage and IPD before vaccination to predict disease impact. We show that the predicted IPD impact closely matches the observed impact in those nine sites and that the model is able to replicate the substantial heterogeneity of impact among sites. We find that a pre-PCV serotype specific case series of IPD (e.g. through sentinel surveillance or surveillance data where the denominator data are unclear), rather than IPD incidence as used in previous approaches, and a cross-sectional carriage study, provide sufficient data to predict the percentage reduction in IPD after maturity of the PCV programme has been reached. If supplemented by pre-PCV pneumococcal disease incidence data, the absolute impact of PCV can be estimated. This study for the first time uses the observed impact of PCV7 from multiple sites to rigorously test the predictive ability of the model while showing that differences in schedule, coverage and the use of catch-up campaigns are unlikely to have had a major contribution to the observed heterogeneity in PCV7 impact on IPD. However we cannot fully rule out that other factors, including changes in testing practices and antibiotic usage, have contributed to the heterogeneity in IPD impact among settings.

The methodology employed here bases predictions on the combined information of serotype distribution in IPD and carriage prior to vaccination. It is essential that these data come from the same or well matched populations for the prediction to be valid. While data on IPD usually result from routine surveillance of a defined population which ideally is representative of the epidemiology of IPD in the country, nasopharyngeal carriage surveys may be conducted among a group that may not be representative of the population studied for IPD. If the serotype distribution in the carriage study population is not representative of that in the disease surveillance population the resulting estimate can be misleading. Similarly, if serotypes that are hardly observed in carriage (serotype 1) or epidemic serotypes (serotypes 1 or 5) contribute substantially to the local IPD burden the performance of the model may be impaired. Here we test the predictive ability of the model using IPD surveillance data from large geographic regions paired with data on nasopharyngeal carriage from relatively small population samples of the same geographic regions. We find that in instances where carriage estimates are available from different population subgroups than that of the IPD surveillance population (see Table 1) there was only limited heterogeneity between them suggesting that those samples were representative of the carriage epidemiology in the country. However, the pneumococcal serotypes are affected by secular changes [27] which could also result in a temporal mismatch of carriage and IPD data. The impact of PCV on IPD incidence in Greece and Canada was only reported in Crete and Calgary (IRR 0.61 (0.06 to 6.50) and 0.56 (0.22 to 1.42)) for which no carriage data was available. However, carriage data were available from other regions of Greece and Canada [28–31]. If used to predict the impact of PCV7 we estimated a likely IRR of 0.29 (0.00 to 1.49) and 0.46 (0.20 to 0.78) for Greece and Canada respectively assuming full serotype replacement.

Our predictions are formed on the grounds of three major assumptions: (i) that vaccine serotypes will eventually be eliminated in the post vaccination era, (ii) that VT carriage is replaced by NVT carriage to a proportion *λ =* 1 (complete replacement) and (iii) that the average pathogenicity of non-vaccine serotypes remains unchanged after vaccination. In most settings almost complete elimination of both vaccine serotype carriage and disease has been observed [9–12] and the competition of NVTs has a potential supporting role in this which could help elimination even under low coverage or high infection pressure [19,32,33]. However, evidence that vaccine serotypes are eliminated in a mature programme is still sparse in low- and middle-income settings and it is yet unclear if in settings with high transmission PCVs will still induce sufficient herd protection to interrupt VT transmission. Hence by assuming elimination of VT carriage and disease in our model we predict the potentially vaccine preventable burden when accounting for serotype replacement. We explore the prediction for scenarios without serotype replacement and with complete serotype replacement and use the latter as the most likely scenario which we validated against the observed impact of PCV7 on childhood IPD. While full serotype replacement has been frequently reported from carriage surveys in mature pneumococcal conjugate vaccination programmes [34,35] not all studies fully support this finding [10]. The presented model provides the flexibility to explore deviating assumptions on the proportion to which NVT replace VT in carriage. The average pathogenicity of the NVT group can change following vaccination if NVT serotypes of high pathogenicity disproportionally replace compared with NVT serotypes of lower pathogenicity as was observed following PCV7 when there was a substantial rise in both carriage and disease due to serotype 19A. However, the carriage prevalence rank order of serotypes were found to be associated with the size of their capsule and therefore is thought to be generally stable [36] and to increase proportionally after vaccination [14]. Furthermore the invasive potential of serotypes has found to be a globally stable property which is also constant with time [37–39].

We excluded Norway from the analysis where unlike any other setting a 40% reduction in post vaccination NVT IPD was reported [12]. Pre-PCV7 carriage information was reported from two sites in Norway leading to a pooled estimate for the proportion of VT among carriers of 0.52 (0.22 to 0.80) [40,41]. From the serotype distribution in both carriage and IPD we predict an IRR for all-serotype IPD of 0.52 (0.31 to 1.21) in a mature programme. By contrast, Feikin et al. estimate 0.17 (0.13 to 0.23) from surveillance data (S5 Fig). However, the estimate for the observed impact is based on the extrapolation of a pre vaccination increase in IPD which runs the potential risk of overestimation of the vaccine impact [42]. Without accounting for pre vaccination trends the IRR from routine IPD surveillance is estimated at 0.46 (0.32 to 0.65), as was estimated elsewhere [12,43], and is in line with our prediction. While only in Norway a significant reduction of NVT IPD in children was estimated it may reflect the inherent limitations of an ecological design to estimate the impact of vaccination after the start of routine vaccination; its susceptibility to other factors that impact on IPD incidence. We further studied the sensitivity of the models predictive value to the delay between the start of vaccination and the impact estimate from surveillance. As our base case we presented all analysis in comparison to the observed IRR three years after the introduction of PCV7. Three years were chosen as a trade-off between allowing sufficient time for herd effects and serotype replacement to stabilise and including as many data sets in the analysis as possible. We find that the predictive ability of our model is not affected by the choice of longer post vaccination periods (S6 Fig).

As estimates of PCV7 impact from low- and middle-income countries are only recently becoming available, we could only validate our method against the use of PCV7 in high-income countries and its effects in children. The validity of our results should hold for other pneumococcal disease endpoints, including non-bacteraemic pneumonia, other age groups, in particular the elderly, and conjugate vaccine formulations of higher valency; data to validate this expectation are only recently becoming available. The importance of carriage data to estimate the dynamic effects of vaccination has been increasingly recognised [44]. However, still only limited information on nasopharyngeal carriage in older age groups, particularly in elderly is collected [24] and in many settings no carriage information is available.

The potential importance of pneumococcal carriage for supporting the licensing of pneumococcal conjugate vaccines has recently been outlined [44]. We add to this by making the case for data on pneumococcal carriage to supplement that on IPD for predicting the likely impact of PCV vaccination. We present evidence that the heterogeneity in the observed impact of PCV7 on IPD is largely due to a combination of carriage and IPD of VTs and that other factors including vaccine schedule, coverage or the use of catch up campaigns have a minor role. This method could prove useful to assess the potential impact of future conjugate vaccine formulations, aid with the impact assessments of PCVs into countries where population based surveillance of IPD is not possible, and provide an impact prediction tool to countries who have not yet introduced PCV. With a growing body of evidence on the impact of different PCV formulation and from low income countries further validation will be essential to determine the full potential of this simple model.

## Supporting Information

S1 Fig Literature review flow diagram, (TIFF)

S2 Fig Posterior density distributions of the meta analysis on the proportion of VT carriers. (TIFF)

S3 Fig Association of observed IRR with schedule, coverage and catch up campaigns (I). Observed IRRs stratified into common schedules (top panels), coverage levels (middle panels) and catch up campaigns (lower panels). Error bars present confidence intervals as reported by Feikin et al [12].
(TIFF)

S4 Fig Association of observed IRR with schedule, coverage and catch up campaigns (II). Comparison of the marginal distributions of IRRs in settings with the same schedule (top panel), same coverage levels (middle panel) and that similarly used a catch-up campaign for introduction (lower panel).
(TIFF)

S5 Fig Sensitivity analysis including Norway. Comparison of predicted and observed impact of PCV7 on IPD in children younger than 5 years assuming no serotype replacement (upper panel) or full serotype replacement (lower panel).
(TIF)

S6 Fig Sensitivity analysis including longer time-frames. Comparison of predicted and observed impact (3, 4, 5 years after the introduction of PCV7, from left to right) of PCV7 on IPD in children younger than 5 years assuming full serotype replacement.
(TIF)

S1 Code Model code. (R)
