# Supplementary figures and images for: The Serotype Distribution among Healthy Carriers before Vaccination Is Essential for Predicting the Impact of Pneumococcal Conjugate Vaccine on Invasive Disease
Source: PLoS Comput Biol. 2015 Apr 16;11(4):e1004173. doi: 10.1371/journal.pcbi.1004173 (PMC4400047; doi:10.1371/journal.pcbi.1004173)

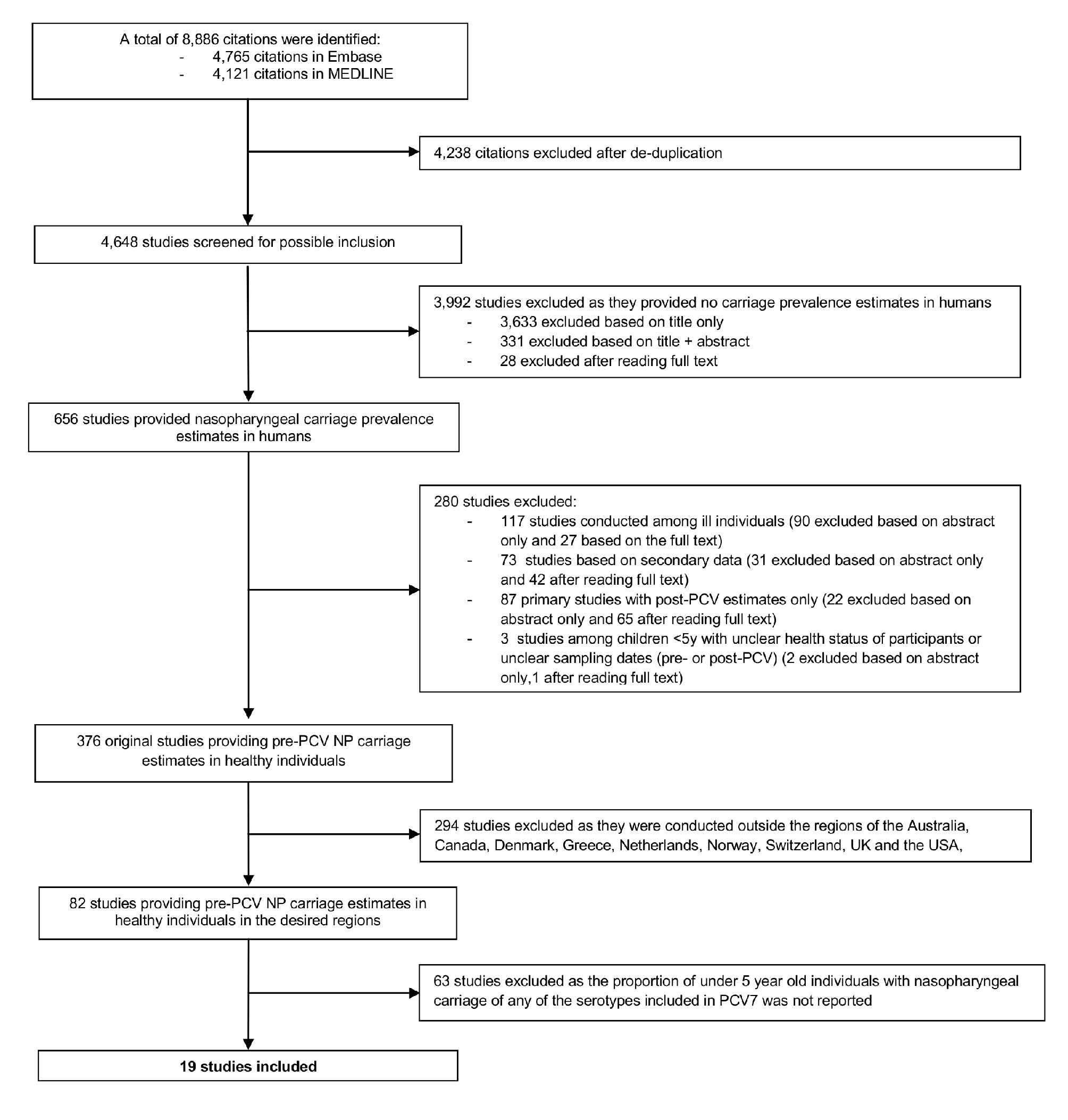

Supplement: S1 Fig — (TIFF) [file pcbi.1004173.s001.tiff]

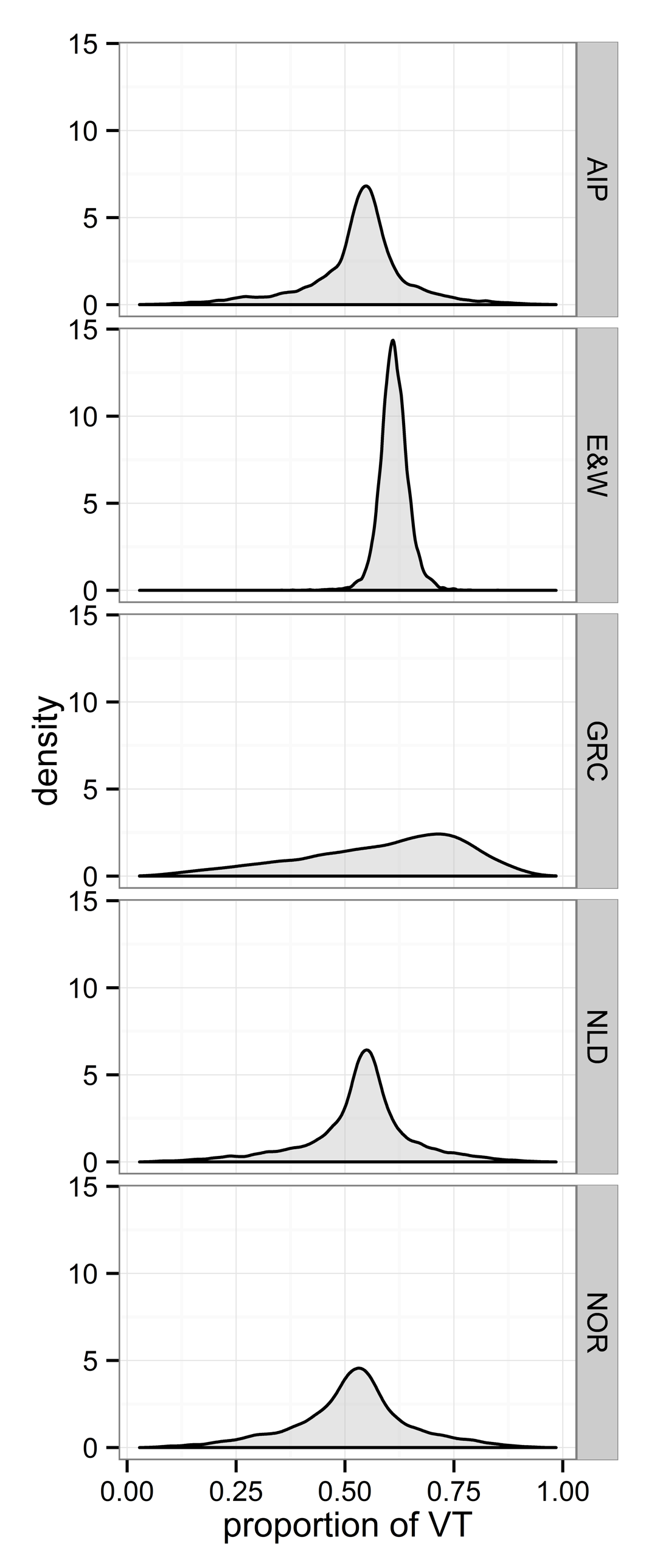

Supplement: S2 Fig — (TIFF) [file pcbi.1004173.s002.tiff]

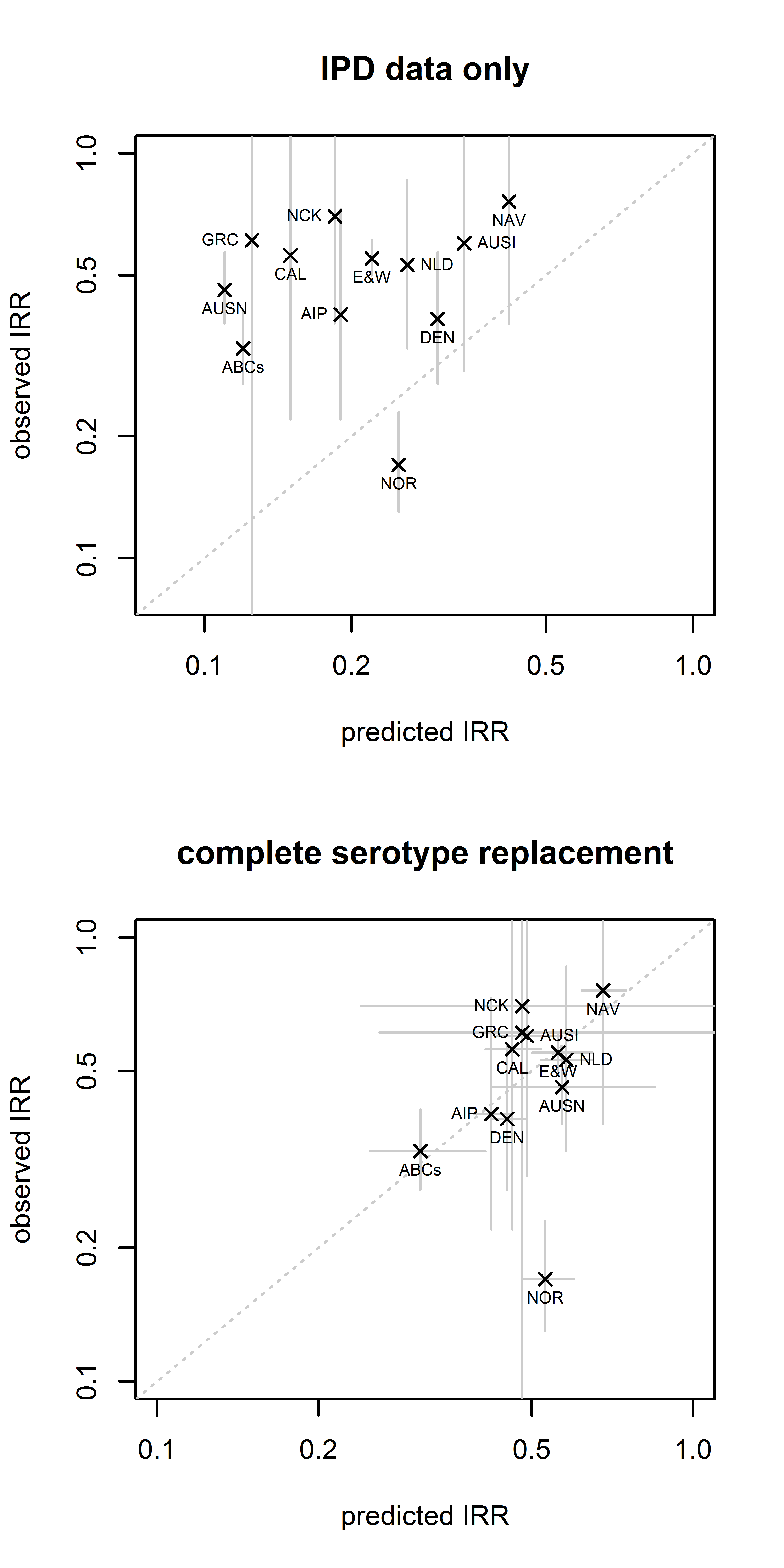

Supplement: S5 Fig — Comparison of predicted and observed impact of PCV7 on IPD in children younger than 5 years assuming no serotype replacement (upper panel) or full serotype replacement (lower panel). (TIF) [file pcbi.1004173.s005.tif]

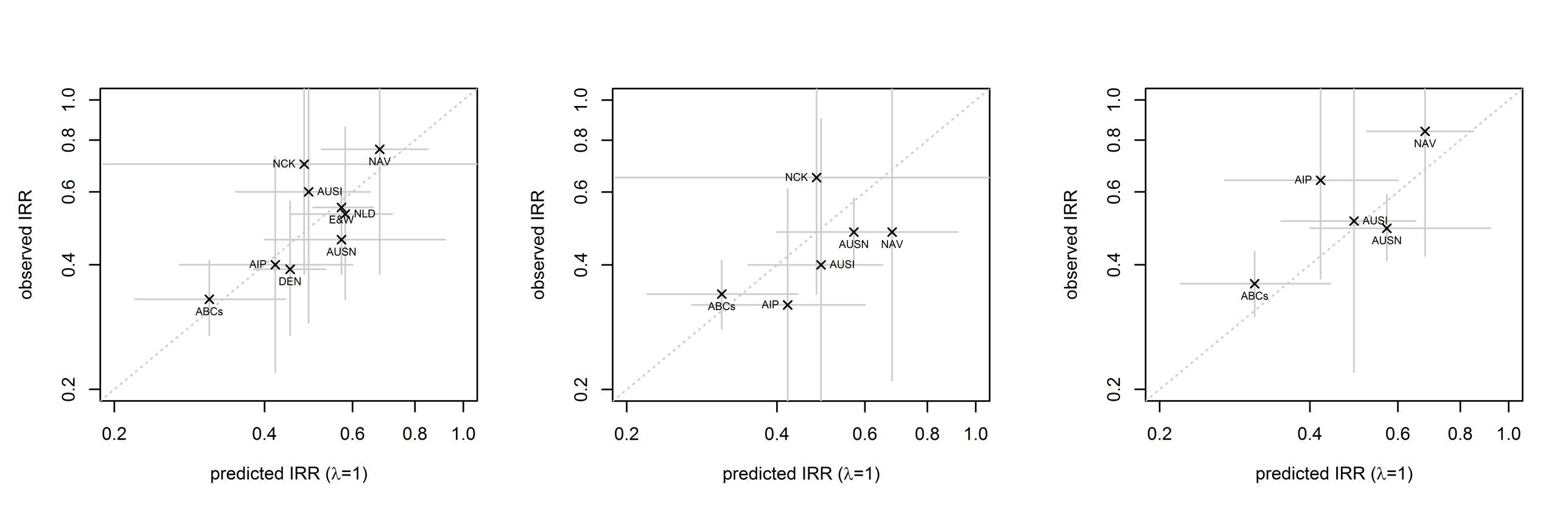

Supplement: S6 Fig — Comparison of predicted and observed impact (3, 4, 5 years after the introduction of PCV7, from left to right) of PCV7 on IPD in children younger than 5 years assuming full serotype replacement. (TIF) [file pcbi.1004173.s006.tif]
